# Author Correction: The LipoGlo reporter system for sensitive and specific monitoring of atherogenic lipoproteins

**DOI:** 10.1038/s41467-020-17532-7

**Published:** 2020-07-21

**Authors:** James H. Thierer, Stephen C. Ekker, Steven A. Farber

**Affiliations:** 1 Carnegie Institution for Science Department of Embryology, 3520 San Martin Drive, Baltimore, MD 21218 USA; 2 Johns Hopkins University Department of Biology, 3400N Charles Street, Baltimore, MD 21218 USA; 3 Department of Biochemistry and Molecular Biology, Mayo Clinic, 200 First Street SW, Rochester, MN 55905 USA

**Keywords:** Atherosclerosis, Imaging

Correction to: *Nature Communications* 10.1038/s41467-019-11259-w, published online 31 July 2019.

The original version of this Article contained errors in Fig. 3 and in the Source data file, for which the authors apologise. In Fig. 3c, the gels representing *mtp*^−/−^ and *apoC2*^−/−^ were switched, and the labels on the left of this panel were incorrectly positioned; moreover, in Fig. 3f and g, the chart titles were omitted. In the Source data file for Fig. 3f, the values for the 5-dpf and 6-dpf wild-type samples were identical. These errors originated when the data from the original files were compiled into the source data and did not affect any figures or quantifications.

The errors have been corrected in both the PDF and HTML versions of the Article, and the Source Data file has been amended to include the correct values for the samples corresponding to Fig. 3f.

