# Supplementary material for: The LipoGlo reporter system for sensitive and specific monitoring of atherogenic lipoproteins
Source: Nat Commun. 2019 Jul 31;10:3426. doi: 10.1038/s41467-019-11259-w (PMC6668417; doi:10.1038/s41467-019-11259-w)
Supplement: Supplementary file 1 — Supplementary Information [file 41467_2019_11259_MOESM1_ESM.pdf]

Supplementary Information

The LipoGlo reporter system for sensitive and specific monitoring of atherogenic lipoproteins

Thierer et al.

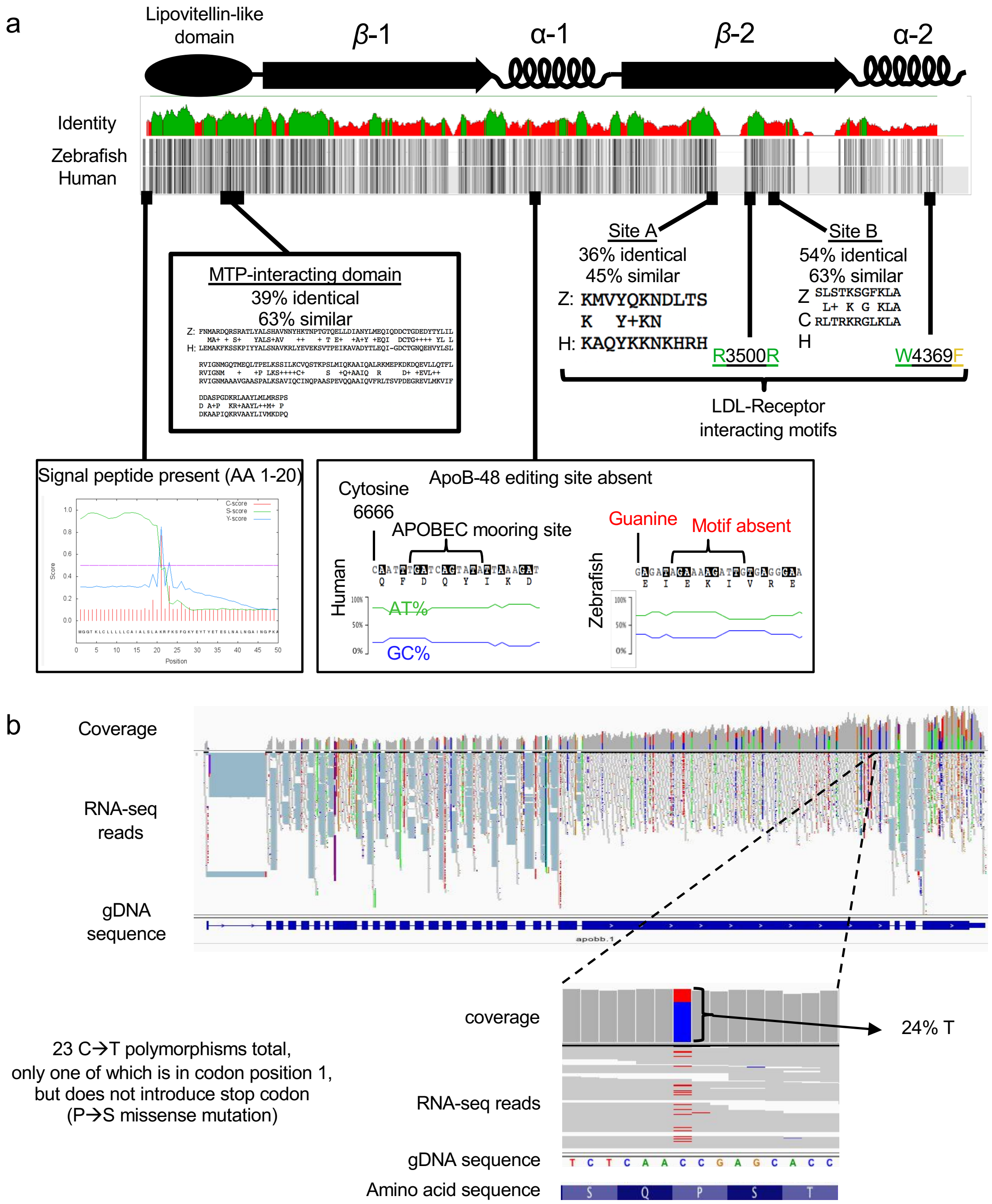

Supplementary Figure 1: Conservation of functional domains in the zebrafish *apoBb.1* ortholog of Human *APOB*. (a) APOB has a penta-partite domain structure, with an amino-terminal globular domain followed by a series of beta and alpha domains. Consistent with other apolipoprotein sequences, APOB shows relatively low sequence conservation between species at the amino acid level (25% identical, 43% similar, green indicates >30% identity in identity plot). However, sequence conservation is enriched in known ApoB functional domains. For example, there is clear conservation of a signal peptide motif at the amino terminus. The MTP-interacting domain shows 39% identity and 63% similarity, and the LDL-R interacting motifs are also well-conserved. However, the *APOB-48* editing site appears completely absent, as zebrafish *apoBb.1* lacks the essential C6666 that is edited to form the premature stop, as well as the APOBEC mooring site, and shows only mild AT-richness that has been shown to be important for APOBEC binding **(b)** To further evaluate whether *apoB*-editing takes place in zebrafish, RNA reads were mapped back to this genomic locus. Post-transcriptional C→U editing would appear as a C→T polymorphism in the genomic sequence. 23 instances of C→T polymorphism were observed, but the vast majority (21) appeared in the wobble position (position 3) of the codon as would be expected for true polymorphisms (rather than post-transcriptional RNA-editing). Of the single instance that occurred in position 1, this did not result in a premature stop codon, providing further support for the absence of APOB-editing activity in zebrafish.

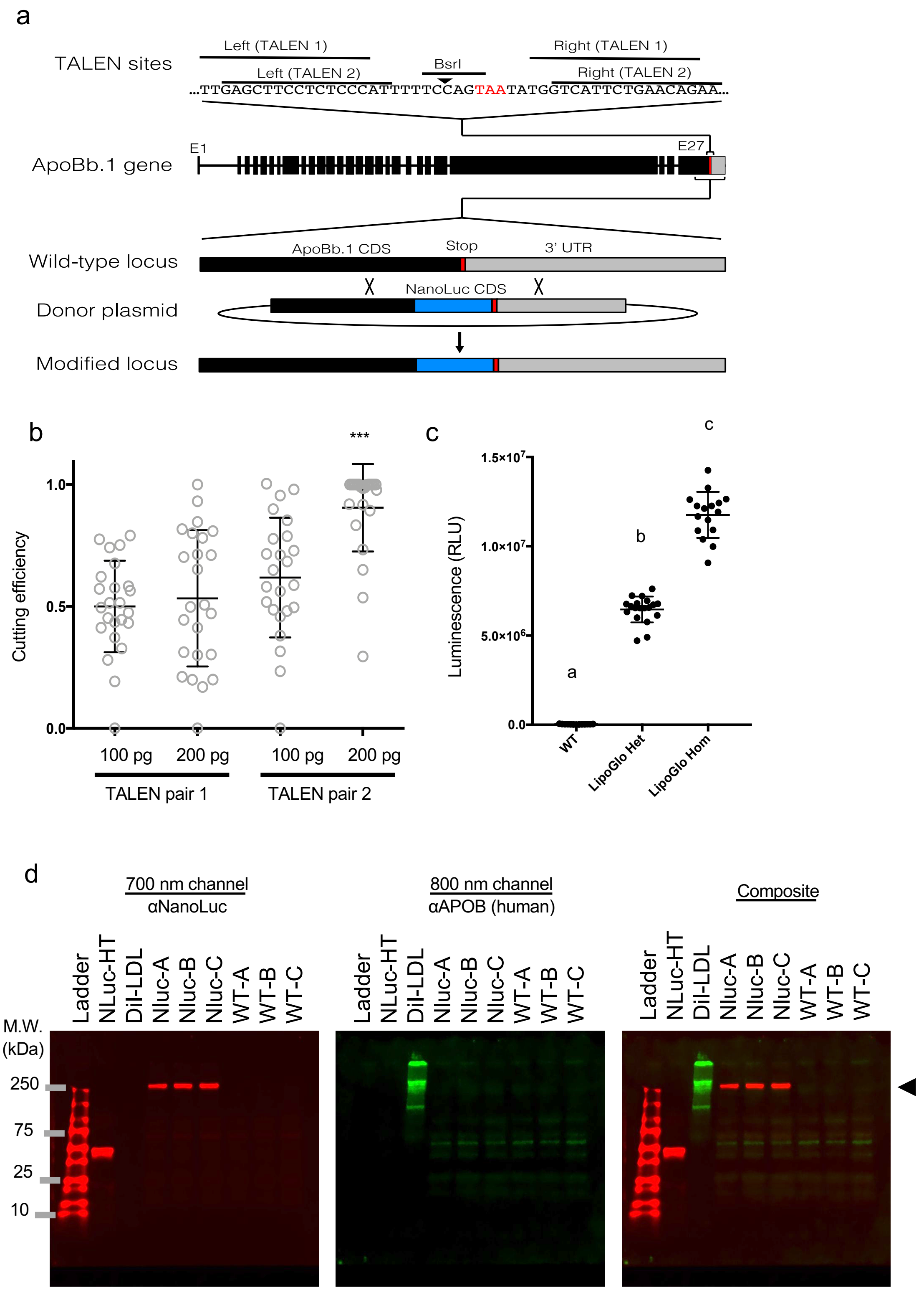

Supplementary Figure 2: Introduction of an in-frame NanoLuc fusion reporter at the endogenous *apoBb.1* locus. (a) A BsrI restriction site overlaps partially with the *apoBb.1* stop codon. Two independent pairs of TALENs were designed as shown, and **(b)** tested for cutting efficiency which was quantified as a loss of susceptibility to BsrI digest. TALEN pair 2 showed significantly higher cutting efficiency, and was selected for co-injection with the DNA donor construct (DF=3, n=24, ANOVA $p<0.0001$, Tukey's HSD $p<.0001$). Horizontal lines denote mean and standard error. **(c)** An incross of adult fish heterozygous for the LipoGlo reporter revealed the expected mendelian ratio of offspring, and showed that homozygous carriers produce approximately twice the signal intensity as heterozygotes (1.2E7±1.3E6 vs 6.5E6±7.3E5) (DF=2, n≈16, ANOVA $p<0.0001$, Tukey's HSD $p<.0001$). Horizontal lines denote mean and standard error. **(d)** SDS-PAGE demonstrating that the NanoLuc reporter remains attached to ApoB. Purified halo-tagged NanoLuc protein (Nluc-HT, ~54 kDa) purchased from Promega was used to indicate the approximate migration of free NanoLuc protein, and DiI-LDL was used to mark the migration of APOB. Protein was extracted from wild-type larvae as well as those homozygous for the NanoLuc reporter, separated by SDS-PAGE, and the resulting blot was probed simultaneously for NanoLuc (Red) and Human APOB (Green). NanoLuc is exclusively detectable in a high-molecular weight band (>250 kDa, black arrowhead), corresponding to the migration of ApoB. Note that the anti-ApoB antibody does not recognize zebrafish ApoB. Source data are provided as a Source Data file.

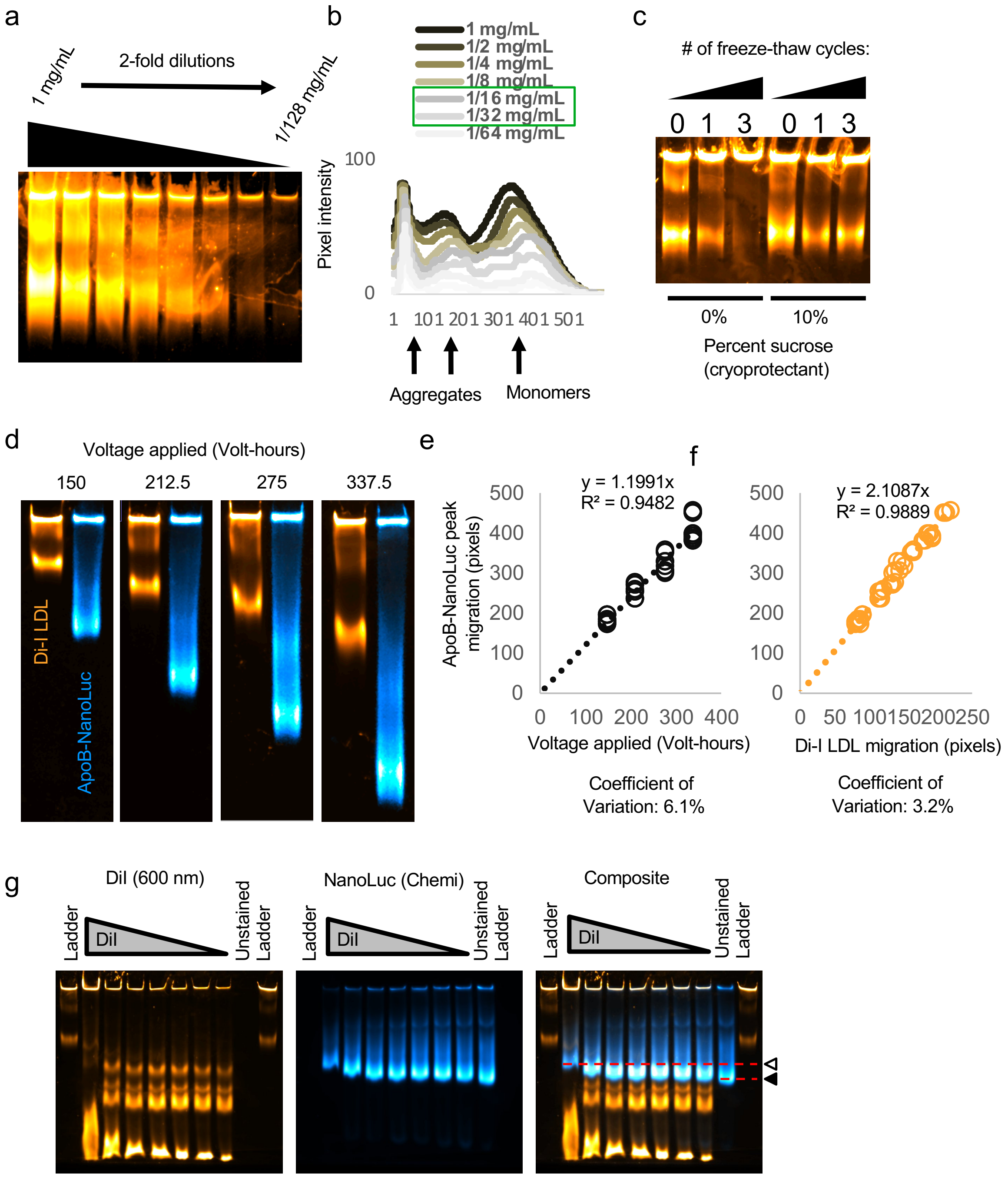

Supplementary Figure 3: Development of an effective migration standard for lipoprotein gels. It is essential that lipoprotein gels include a ladder or normalization standard that has similar electrophoretic properties to ApoB-LPs. Di-I labeled human LDL serves as a commercially available option that enables standardization not only between multiple gels but also between different labs. **(a)** Di-I LDL was subjected to a series of 2-fold dilutions and separated via N-PAGE as described and imaged with the Licor-Fc to determine an appropriate dilution factor that was still readily detectable. **(b)** Plot profiles of each of the serial dilutions revealed retardation of peak mobility in the highly concentrated samples, potentially due to overcrowding. Dilution factors between 16 and 32-fold were selected as acceptable (green box), and a 24-fold dilution was used for subsequent assays. **(c)** Sucrose was included as a cryoprotectant during Di-I LDL dilution, and there is no change in peak particle mobility across at least 3 freeze-thaw cycles in the presence of 10% sucrose, whereas the ladder is almost completely aggregated without cryoprotectant. **(d)** To determine the relationship between mobility of the standard and lipoprotein samples, homogenate was prepared and pooled from *mtp -/-* (3 dpf) mutant larvae (which produce primarily LDL-like particles). Samples of homogenate were run alongside Di-I standard for either 150, 212.5, 275, or 337.5 volt-hours, and the peak migration (in pixels) was quantified for each species. **(e)** While there was a clear linear relationship between ApoB-LP migration and voltage applied (R^2=.95), **(f)** the relationship was much tighter when electrophoretic mobility was compared to the migration standard (R^2=.99), validating the utility of Di-I LDL as a migration standard. **(g)** Plasma extracted from adult female zebrafish was stained with DiI along a series of two-fold dilutions from 30 mg/mL down to approximately .5 mg/mL. Higher concentrations of DiI significantly impeded lipoprotein migration (white arrowhead) relative to unstained plasma (black arrowhead). Source data are provided as a Source Data file.

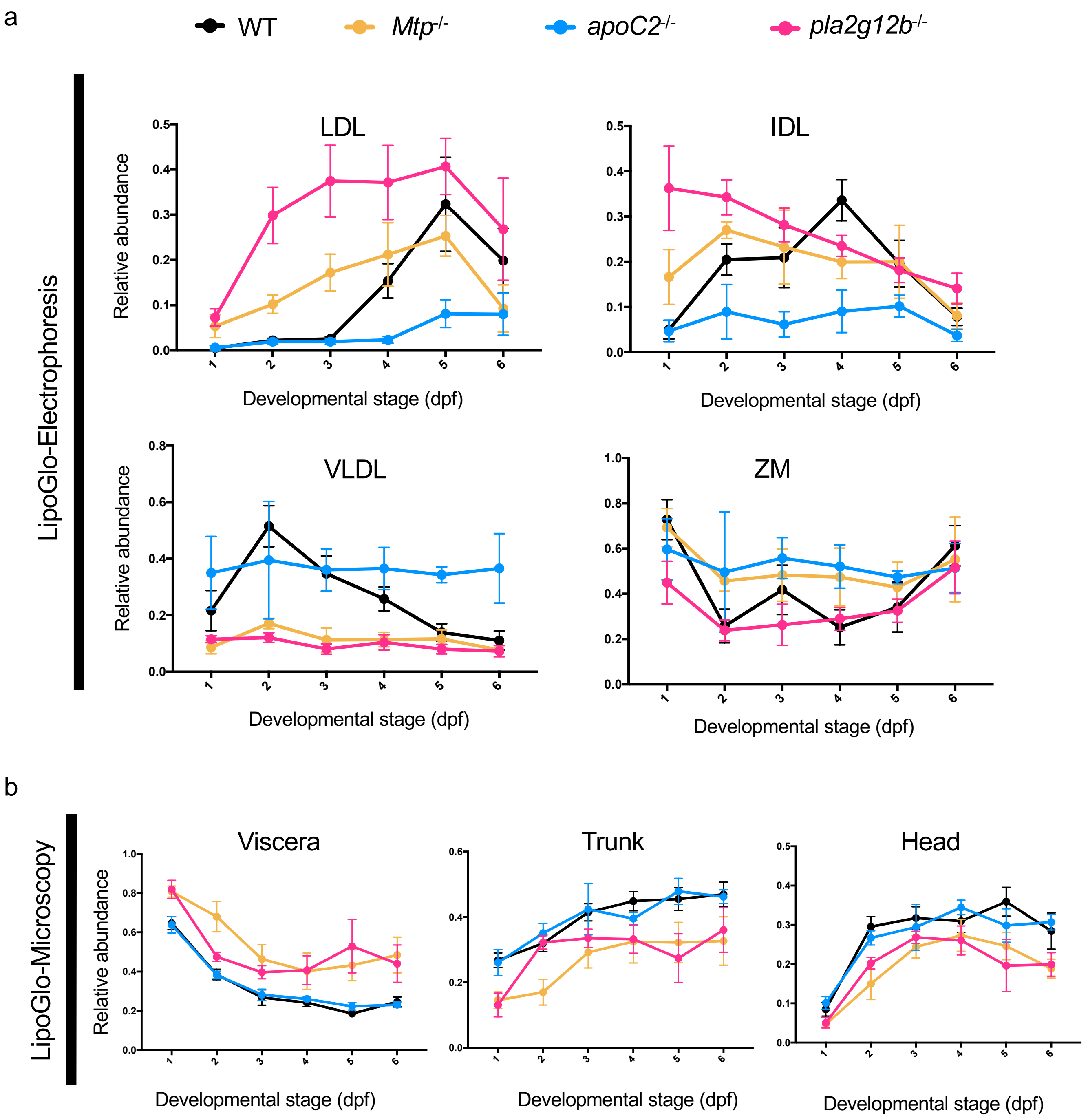

Supplementary Figure 4: Side-by-side analysis of LipoGlo-Electrophoresis and Microscopy results from mutant genotypes. **(a)** Plots of electrophoresis and **(b)** microscopy data reported in the main text grouped by subclass rather than by genotype and showing standard deviations, mean ± SD shown. Source data are provided as a Source Data file.

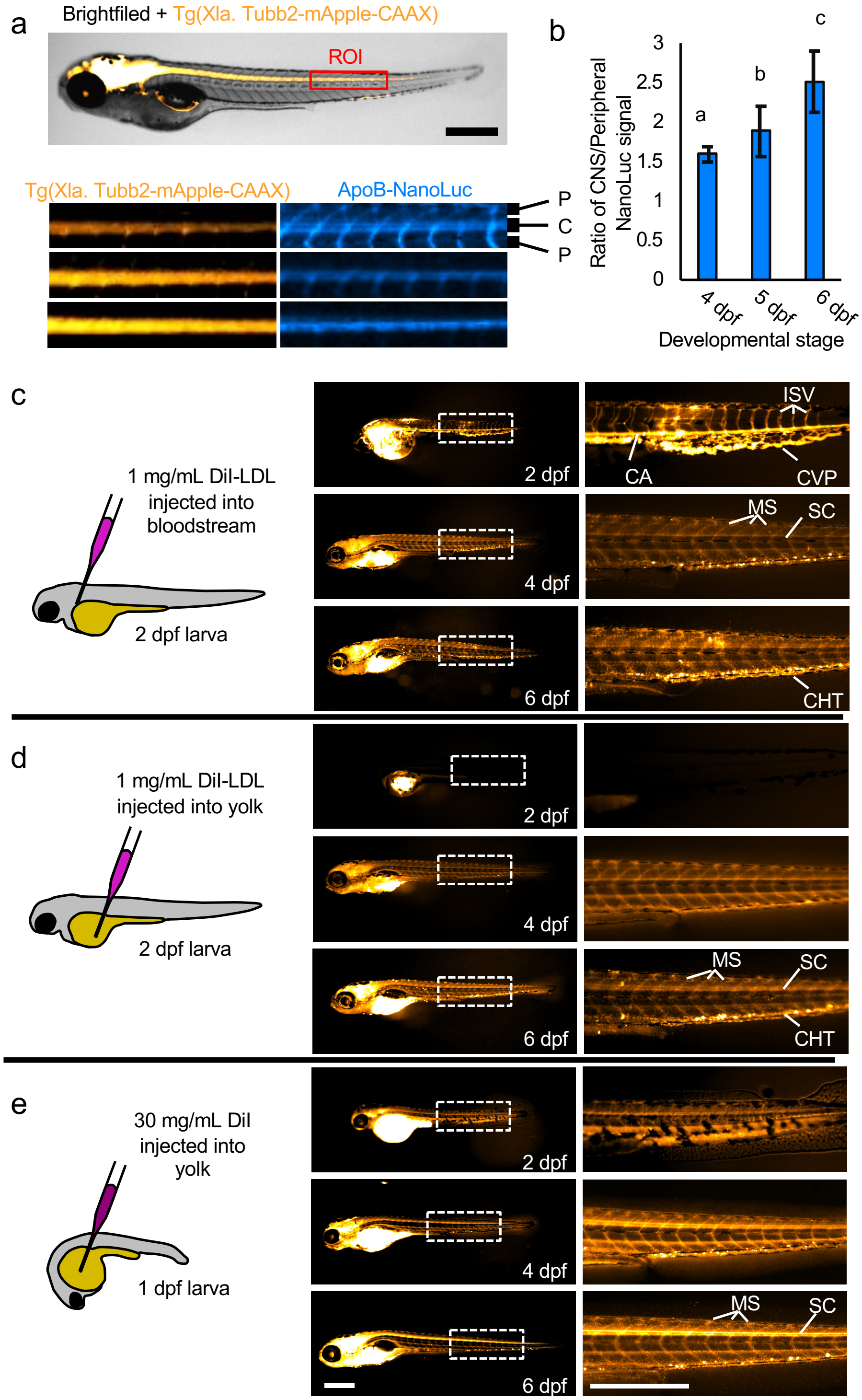

Supplementary Figure 5: LipoGlo microscopy reveals ApoB-LP localization. (a) Three independent clutches of larvae carrying both the CNS marker *Tg(Xla. Tubb2-mApple-CAAX)* and ApoB-NanoLuc fusion were fixed and imaged at 4, 5, and 6 dpf as described in detailed methods. A 20x100 pixel region of interest (ROI) was drawn centered around the spinal cord (marked by mApple) just distal to the intestine. The mApple and ApoB-NanoLuc channels are displayed separately below (representative of 15 images per time point). **(b)** Quantification of the signal intensity in spinal cord (CNS) versus peripheral regions revealed a gradual enrichment of signal in the CNS relative to the periphery from 4-6 dpf (DF=2, n=15, Welch's ANOVA $p<0.0001$, Games-Howell $p<.01$), mean ± SD shown. **(c)** Schematic and results of injection of DiI-labeled human LDL into the common cardinal vein of zebrafish larvae. Immediately following injection on 2 dpf, signal is primarily detectable in blood vessels such as the caudal artery (CA), caudal vein plexus (CVP), and intersegmental vessels (ISV). Later in development (4-6 dpf) signal is primarily localized to extravascular tissues such as the myosepta (MS), spinal cord (SC) and puncta in the caudal hematopoietic tissue (CHT) that likely correspond to macrophages (n=6). **(d)** When DiI-LDL is injected into the yolk, signal is undetectable in the vasculature of early-stage larvae, but in ~50% of cases (3 out of 6) will be detected in both vascular and extravascular tissues of later-stage larvae (n=6). **(e)** A solution of DiI injected into the larval yolk leads to a staining pattern that closely mirrors LipoGlo-microscopy experiments, indicating that it stains endogenous lipoproteins produced in the YSL (N=15). Results represent pooled data from three independent clutches, "n" denotes total number of samples per data point. Scale bars = 500 μm. Source data are provided as a Source Data file.

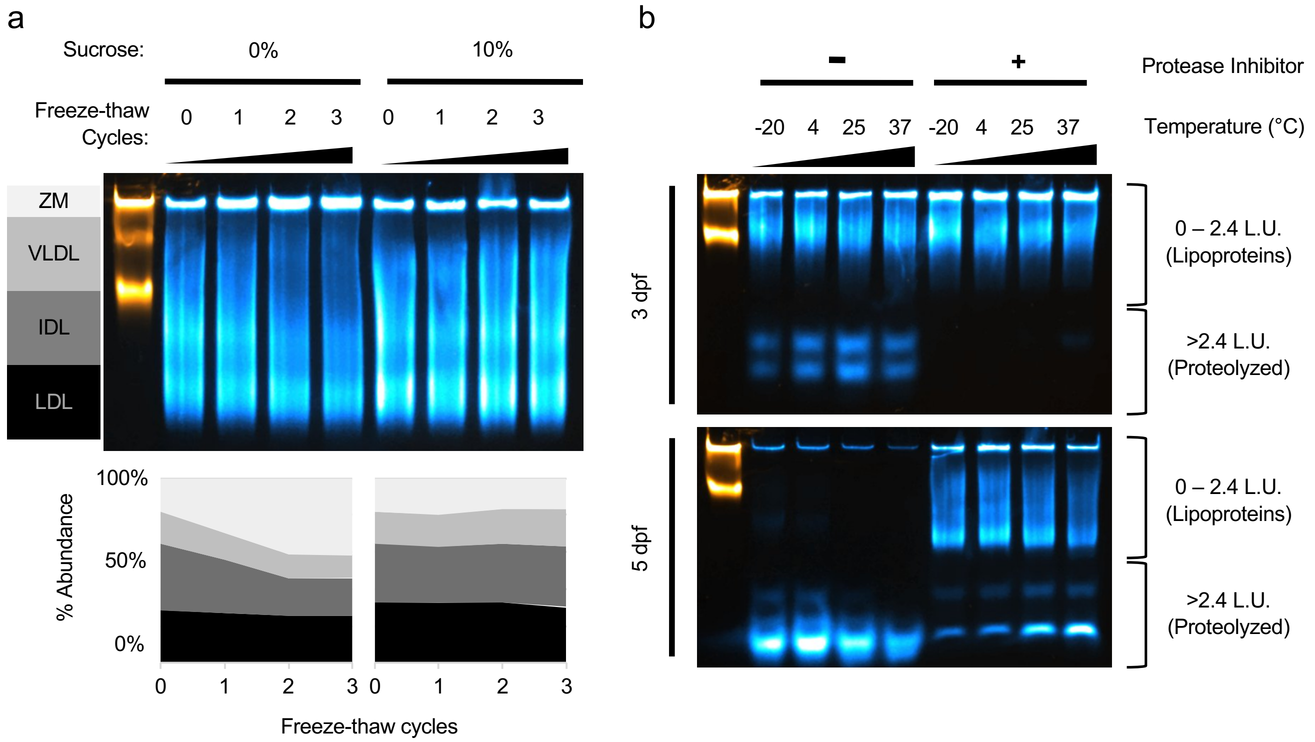

Supplementary Figure 6: Cryoprotectant and protease-inhibition properties of ApoB-LP stabilization buffer. (a) 4 dpf wild-type larvae were homogenized in ApoB-LP stabilization buffer containing 0% or 10% final concentration of sucrose and subjected to between 0 and 3 freeze-thaw cycles, and then separated using native-PAGE as described in detailed methods. While the lipoprotein size distribution remained constant in samples containing sucrose as a cryoprotectant, samples without sucrose showed a gradual enrichment of ZM particles, which appears to be due to aggregation of VLDL and IDL particles. **(b)** Larvae were homogenized in ApoB-LP stabilization buffer with and without the protease inhibitor components (cOmplete mini EDTA-free tablet supplemented with 40 mM final concentration of EGTA, see recipes) and incubated at various temperatures for 2 hours. Samples were then separated by native-PAGE at 50 V for 30 minutes, and 125 V for 60 minutes. This is 125 Volt-hours less than described in detailed methods to enable visualization of proteolysis products. At 3 dpf, protease activity is quite low such that no proteolyzed products are present in the group treated with protease inhibitor, whereas degradation products are visible in a temperature-dependent manner in the absence of inhibitors. By 5 dpf, protease activity is much higher in the homogenate sample, presumably due to development of a mature intestine. Protease activity is still well-controlled in the presence of protease inhibitor at low temperatures, but in the absence of protease inhibitor degradation is so severe that there are signs of both cleavage of NanoLuc from the lipoprotein particle as well as proteolysis of the reporter itself. Source data are provided as a Source Data file.

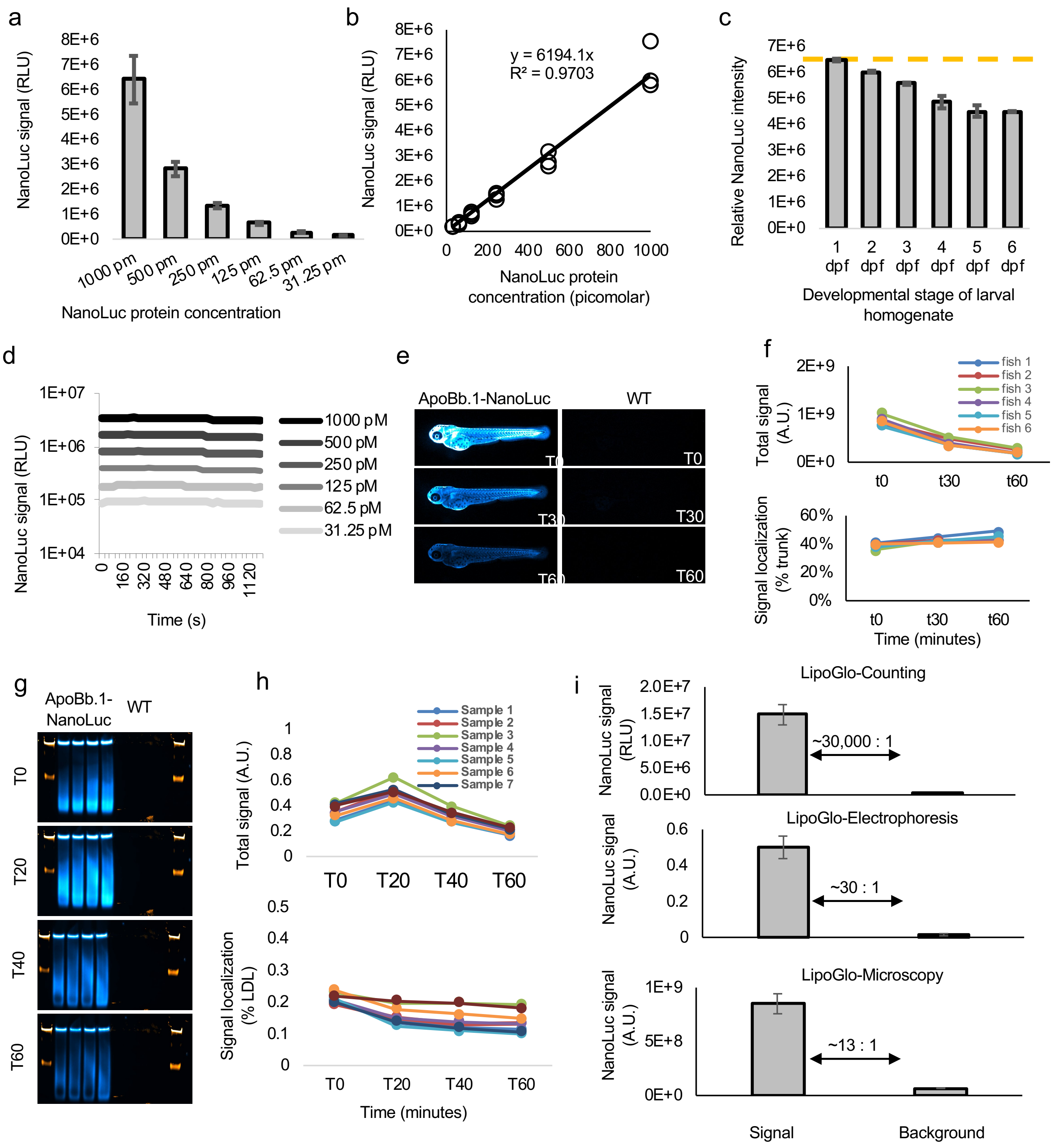

Supplementary Figure 7: NanoLuc standard curves. (a) To determine the absolute concentration of ApoB-LPs in the larval homogenate, purified NanoLuc protein was ordered directly from Promega (Nluc-HT Protein, 500ug, 54.2KDa, #CS188401) and diluted to 1 nM working concentration in 1x ApoB-LP stabilization buffer. This solution was subjected to a 6-point series of 2-fold dilutions and used in a plate-based assay for NanoLuc activity (mean ± SD shown), and **(b)** showed the expected log-linear relationship. **(c)** There is a marked increase in pigmentation throughout larval development, causing homogenate to become progressively more opaque. To test the effect of pigment on NanoLuc readings, wild-type larvae that lack the ApoB-NanoLuc reporter were homogenized in ApoB-LP stabilization buffer at each stage of development. This homogenate was then supplemented with a final concentration of 1 nM NanoLuc protein and subjected to a plate read assay. As expected, the relative intensity of NanoLuc signal declines from 2 – 6 dpf, indicating that absolute quantitation of NanoLuc levels should include a standard curve that accounts for larval pigmentation (mean ± SD shown). **(d)** Analysis of LipoGlo counting signal over time (well values read every 40 seconds for 20 minutes) demonstrated that this assay is robust to slight fluctuations in incubation time, as signal half-lives were calculated to be greater than 60 minutes for all concentrations tested. **(e)** Analysis of LipoGlo microscopy signal over time revealed detectable signal well above background for over 60 minutes, and **(f)** although absolute signal declined over time (top panel), the signal localization remained relatively constant over short fluctuations in incubation time (bottom panel). **(g)** Analysis of LipoGlo electrophoresis signal over time **(h)** revealed that reads immediately after substrate addition (T0) were abnormally low (top panel) and biased toward detection of abundant lipoprotein species (bottom panel). However, following a brief incubation period (minimum of 5 minutes), absolute signal decays gradually over time, but changes in relative signal are much less pronounced (T20 – 60). **(i)** Quantification of the signal to background ratio of each of the LipoGlo assays, mean ± SD shown. Source data are provided as a Source Data file.

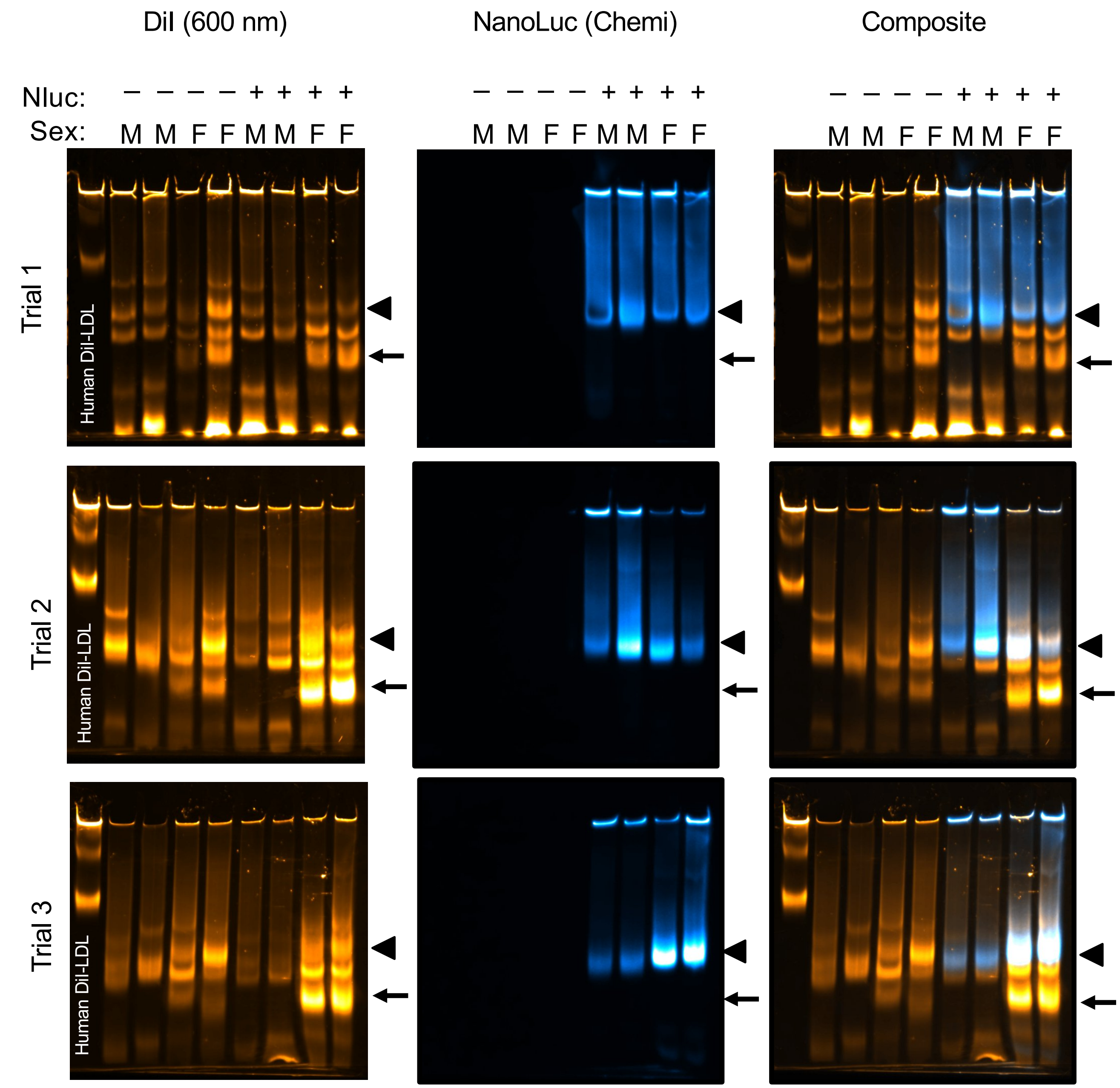

Supplementary Figure 8: Characterization of the lipoprotein profile in adult zebrafish using DiI. Plasma was extracted from adult zebrafish homozygous for the LipoGlo reporter as well as wild-type controls. Plasma was then stained with DiI and then separated via native-PAGE using the LipoGlo-electrophoresis method. Numerous bands appear in the plasma profile in the DiI-channel (600 nm), but the LDL band (black arrowhead) can be identified as the smallest ApoB-positive band using the LipoGlo reporter (chemi channel). The results from three independent trials are shown, with no overt differences detectable between wild-type and LipoGlo animals, although there is significant variation between individuals. One band is present exclusively in the female plasma profile (black arrow), and likely corresponds to vitellogenin, a well-characterized lipid-transport protein that is only expressed in females. Source data are provided as a Source Data file.

Primer #	Purpose	Sequence
1	generate pME NanoLuc F	GGG GAC AAG TTT GTA CAA AAA AGC AGG CTT GAT GGT CTT CAC ACT CGA AGA TTT C
2	generate pME NanoLuc R	GGG GAC CAC TTT GTA CAA GAA AGC TGG GTT TAC GCC AGA ATG CGT TCG CA
3	generate left homology arm F	GGG GAC AAC TTT GTA TAG AAA AGT TGC GCT GCC TGG AAT GAA TGA AGC
4	generate left homology arm R	GGG GAC TGC TTT TTT GTA CAA ACT TGT CTG GAA AAA TGG GAG AGG AAG
5	generate right homology arm F	GGG GAC AGC TTT CTT GTA CAA AGT GGA ATA TGG TCA TTC TGA ACA GAA AGT AAA
6	generate right homology arm R	GGG GAC AAC TTT GTA TAA TAA AGT TGG GTA AGG CAG ACA TCA GTT TGT AAG
7	test TALEN cutting efficiency F	TGC AAT GAA GCA AAT CGA AAG TC
8	test TALEN cutting efficiency R	AAG ATT GGG TCG TGT TGC AT
9	genotype LipoGlo F	GCT TCC TCT CCC ATT TTT CC
10	genotype LipoGlo R1	CCC CGA GAT TCT GAA ACA AAC
11	genotype LipoGlo R2	AAG TGT CCA TTG GCT TCG AT
12	genotype mtp F	GTC TGA GGT TCA GAT GTA CCT GTT AGG AC
13	genotype mtp R	CTC TGC TGT GAT GAG CGC AGG
14	genotype apoc2 F	GAG CGG AGA GCT TTC GTG T
15	genotype apoc2 R	CTT CCA GCT TGT AGC CCT TG
16	genotype pla2g12b F	ACA AGG GAA AGC AAA CCA AA
17	genotype pla2g12b R	CAG TGT TGT ACA TGG TGT CTG C

Supplementary Table 1: Primers used in this study

REAGENT or RESOURCE	SOURCE	IDENTIFIER
Critical Commercial Assays		
Nano-Glo assay	Promega Corp	N1110
Di-I-Labeled fluorescent LDL	ThermoFisher Scientific	L3482
Experimental Models: Organisms/Strains		
ApoB-NanoLuc fusion allele ($apoBb.1^{NLuc}$)	Farber Lab (Carnegie)	ZIRC stock TBD
Mutant mtp allele (mtp^{stl})	Yaniv Lab (Weizmann)	ZIRC stock TBD
Mutant apoC2 allele ($apoC2^{sd38}$)	Miller Lab (UCSD)	ZIRC stock TBD
Mutant pla2g12b allele ($pla2g12b^{sa659}$)	Sanger Institute	ZIRC stock TBD
CNS marker Tg(Xla.Tubb2:mapple-CAAX)	Halpern Lab (Carnegie)	ZIRC stock TBD
Recombinant DNA		
Gateway plasmid with left homology arm sequence pDONR_P4-P1R_5'arm_apobb.1	Farber Plasmid Stock 1494	Addgene stock 128662
Gateway plasmid with nanoluc coding sequence pDONR_221_middle-entry_NanoLuc	Farber Plasmid Stock 1496	Addgene stock 128673
Gateway plasmid with right homology arm sequence pDONR_P2R-P3_3'arm_apobb.1	Farber Plasmid Stock 1495	Addgene stock 128681
Donor plasmid for HDR pDestTol2pA2-LA-nanoLuc-RA	Farber Plasmid Stock 1511	Addgene stock 128692
TALEN pair 1 – left arm pT3TS-TAL-apobb.1-PR1-LA	Farber Plasmid Stock 1514	Addgene stock 128693
TALEN pair 1 – right arm pT3TS-TAL-apobb.1-PR1-RA	Farber Plasmid Stock 1515	Addgene stock 128694
TALEN pair 2 – left arm pT3TS-TAL-apobb.1-PR2-LA	Farber Plasmid Stock 1512	Addgene stock 128695
TALEN pair 2 – right arm pT3TS-TAL-apobb.1-PR2-RA	Farber Plasmid Stock 1513	Addgene stock 128696

Supplementary Table 2: Key resources table
