# Supplementary material for: The LipoGlo reporter system for sensitive and specific monitoring of atherogenic lipoproteins
Source: Nat Commun. 2019 Jul 31;10:3426. doi: 10.1038/s41467-019-11259-w (PMC6668417; doi:10.1038/s41467-019-11259-w)
Supplement: Supplementary file 3 — Description of Additional Supplementary Files [file 41467_2019_11259_MOESM3_ESM.docx]

**Description of Supplementary Files**

**File Name:** **Supplementary Software 1**

**Description:** Supplementary Software 1 is comprised of an excel sheet that contains instructions and templates for analyzing LipoGlo-electrophoresis data in a semi-automated and reproducible manner.
