# Supplementary material for: The LipoGlo reporter system for sensitive and specific monitoring of atherogenic lipoproteins
Source: Nat Commun. 2019 Jul 31;10:3426. doi: 10.1038/s41467-019-11259-w (PMC6668417; doi:10.1038/s41467-019-11259-w)
Supplement: Supplementary file 5 — Reporting Summary [file 41467_2019_11259_MOESM5_ESM.pdf]

nature research

Corresponding author(s): Steven A. Farber

Last updated by author(s): 2019/05/16

Reporting Summary

Nature Research wishes to improve the reproducibility of the work that we publish. This form provides structure for consistency and transparency in reporting. For further information on Nature Research policies, see Authors & Referees and the Editorial Policy Checklist.

Statistics

For all statistical analyses, confirm that the following items are present in the figure legend, table legend, main text, or Methods section.

n/a	Confirmed	
☐	☒	The exact sample size (*n*) for each experimental group/condition, given as a discrete number and unit of measurement
☐	☒	A statement on whether measurements were taken from distinct samples or whether the same sample was measured repeatedly
☐	☒	The statistical test(s) used AND whether they are one- or two-sided<br>*Only common tests should be described solely by name; describe more complex techniques in the Methods section.*
☒	☐	A description of all covariates tested
☐	☒	A description of any assumptions or corrections, such as tests of normality and adjustment for multiple comparisons
☐	☒	A full description of the statistical parameters including central tendency (e.g. means) or other basic estimates (e.g. regression coefficient) AND variation (e.g. standard deviation) or associated estimates of uncertainty (e.g. confidence intervals)
☐	☒	For null hypothesis testing, the test statistic (e.g. *F*, *t*, *r*) with confidence intervals, effect sizes, degrees of freedom and *P* value noted<br>*Give P values as exact values whenever suitable.*
☒	☐	For Bayesian analysis, information on the choice of priors and Markov chain Monte Carlo settings
☒	☐	For hierarchical and complex designs, identification of the appropriate level for tests and full reporting of outcomes
☒	☐	Estimates of effect sizes (e.g. Cohen's *d*, Pearson's *r*), indicating how they were calculated

Our web collection on statistics for biologists contains articles on many of the points above.

Software and code

Policy information about availability of computer code

Data collection	No software was used in data collection.
Data analysis	A custom excel sheet contains formulas that expedite data analysis, and is provided as supplementary software 1.

For manuscripts utilizing custom algorithms or software that are central to the research but not yet described in published literature, software must be made available to editors/reviewers. We strongly encourage code deposition in a community repository (e.g. GitHub). See the Nature Research guidelines for submitting code & software for further information.

Data

Policy information about availability of data

All manuscripts must include a data availability statement. This statement should provide the following information, where applicable:

- Accession codes, unique identifiers, or web links for publicly available datasets
- A list of figures that have associated raw data
- A description of any restrictions on data availability

All data is available upon request.

Field-specific reporting

Please select the one below that is the best fit for your research. If you are not sure, read the appropriate sections before making your selection.

☒ Life sciences ☐ Behavioural & social sciences ☐ Ecological, evolutionary & environmental sciences

For a reference copy of the document with all sections, see nature.com/documents/nr-reporting-summary-flat.pdf

Life sciences study design

All studies must disclose on these points even when the disclosure is negative.

Sample size	No prior information was available to estimate sample size, as lipoproteins have not previously been characterized in zebrafish larvae. Sample sizes were designed to be as large as reasonably possible while maintaining sibling controls from the same clutch.
Data exclusions	Several samples were not quantified for the LipoGlo imaging experiment as a result of sample destruction during processing. All quantified samples were included in analyses.
Replication	All key experiments were replicated three times from independent clutches to verify reproducibility.
Randomization	Randomization was not relevant for the majority of experiments described, as groups were allocated based on genotype. In the case of drug treatment experiments, random individuals were selected for drug or vehicle treatment from within the same clutch.
Blinding	Blinding was not relevant to this study, as experiments provided quantitative readouts (e.g. plate read data) that are not susceptible to biases from the experimenter.

Reporting for specific materials, systems and methods

We require information from authors about some types of materials, experimental systems and methods used in many studies. Here, indicate whether each material, system or method listed is relevant to your study. If you are not sure if a list item applies to your research, read the appropriate section before selecting a response.

Materials & experimental systems

n/a	Involved in the study	
☒	☐	Antibodies
☒	☐	Eukaryotic cell lines
☒	☐	Palaeontology
☐	☒	Animals and other organisms
☒	☐	Human research participants
☒	☐	Clinical data

Methods

n/a	Involved in the study	
☒	☐	ChIP-seq
☒	☐	Flow cytometry
☒	☐	MRI-based neuroimaging

Animals and other organisms

Policy information about studies involving animals; ARRIVE guidelines recommended for reporting animal research

Laboratory animals	All genotypes were bred into the wild-type AB background. All assays were performed on larvae heterozygous for the ApoB-Nanoluc reporter unless otherwise noted. To monitor the wild-type lipoprotein profile throughout larval development, pairwise crosses were set up between wild-type AB adults and adults homozygous for the ApoB-NanoLuc reporter (apoBb.1NLuc/NLuc). To characterize the lipoprotein profile of mtp mutant larvae 34, pairwise crosses were set up between mtpstl/+ and mtpstl/+ ; apoBb.1NLuc/NLuc adults. To characterize the lipoprotein profile of apoC2 mutant larvae, pairwise crosses were set up between apoC2sd38/sd38 and apoC2sd38/+ ; apoBb.1NLuc/+ adults and larvae positive for the NanoLuc reporter were selected for analysis. To characterize the lipoprotein profile of pla2g12b mutant larvae, pairwise crosses were set up between pla2g12bsa659/sa659 and pla2g12bsa659/+ ; apoBb.1NLuc/+ adults. To evaluate association between the ApoB-LPs and the central nervous system, adults homozygous for the ApoB-NanoLuc reporter (apoBb.1NLuc/NLuc) were crossed to adults heterozygous for the central nervous system marker Tg(Xla.Tubb2:mapple-CAAX), and embryos were screened for mApple prior to fixation and mounting (unpublished reagent provided by the Halpern Lab, c583). As zebrafish sex cannot be determined during the larval stages, gender can be excluded as a variable. Larvae used for this study were between 1 and 15 days post-fertilization.
Wild animals	This study did not involve wild animals.
Field-collected samples	This study did not involve animals collected from the field.
Ethics oversight	All procedures were approved by the Carnegie Institution Animal Care and Use Committee (Protocol #139).

Note that full information on the approval of the study protocol must also be provided in the manuscript.
